# Pembrolizumab Improves Outcomes in Early-Stage and Locally Advanced or Metastatic Triple-Negative Breast Cancer

**DOI:** 10.1093/oncolo/oyac014

**Published:** 2022-03-28

**Authors:** Anne Jacobson

## Abstract

Updates from the phase III KEYNOTE-522 and KEYNOTE-355 trials demonstrate the benefits of pembrolizumab added to chemotherapy in early-stage and locally advanced or metastatic triple-negative breast cancer, respectively.

Pembrolizumab targets the programmed death-1/programmed death-ligand 1 (PD-1/PD-L1) signaling pathway across a range of tumor types with varying degrees of PD-L1 expression. The phase III KEYNOTE-522 and KEYNOTE-355 trials evaluated the addition of pembrolizumab to standard chemotherapy in patients with early-stage and locally advanced or metastatic triple-negative breast cancer (TNBC), respectively ^1, 2^. Updated findings provide insights on the optimal role of pembrolizumab across TNBC treatment settings.

## KEYNOTE-522: Pembrolizumab in Early-Stage TNBC

Peter Schmid, M.D., Ph.D., of Barts Cancer Institute, presented findings from KEYNOTE-522 showing a 37% improvement in event-free survival (EFS) associated with neoadjuvant and adjuvant pembrolizumab in early-stage TNBC ^1^.

The phase III KEYNOTE-522 trial enrolled 1,174 patients with newly diagnosed, operable, stage II-III TNBC. Patients were randomly assigned 2:1 to receive both neoadjuvant and adjuvant therapy with pembrolizumab 200mg every 3 weeks (*n* = 784) or placebo (*n* = 390).

During the neoadjuvant phase of the trial, patients received pembrolizumab or placebo in addition to chemotherapy with carboplatin plus paclitaxel for 12 weeks, followed by doxorubicin or epirubicin plus cyclophosphamide for an additional 12 weeks. Following surgery, patients received 9 additional cycles of adjuvant pembrolizumab or placebo. The co-primary endpoints were pathologic complete response and EFS.

Pembrolizumab added to neoadjuvant and adjuvant therapy was associated with a statistically significant and clinically meaningful 37% improvement in EFS (HR, 0.63; *p* =.00031) (Table 1). The benefit of pembrolizumab was consistent across subgroups, including those defined by disease stage and nodal status.

**Table 1.** Event-free survival by neoadjuvant and adjuvant therapy in early-stage TNBC

Endpoint	Pembrolizumab (*n* = 784)	Placebo (*n* = 390)	HR (95% CI)	*p* value
EFS events	15.7%	23.8%	0.63 (0.48-0.82)	.00031
EFS at 36 months	84.5%	76.8%		

Abbreviations: CI, confidence interval; EFS, event-free survival; HR, hazard ratio; TNBC, triple-negative breast cancer.

Results from the KEYNOTE-522 trial support the role of pembrolizumab plus platinum-based chemotherapy in the neoadjuvant setting, followed by adjuvant pembrolizumab after surgery, as a new standard of care for patients with early-stage TNBC.

## KEYNOTE-355: Pembrolizumab in Locally Advanced or Metastatic TNBC

Javier Cortes, M.D., Ph.D., of the International Breast Cancer Center, presented results from KEYNOTE-355 demonstrating the optimal PD-L1 expression threshold for pembrolizumab benefit in advanced or metastatic TNBC ^2^.

The phase III KEYNOTE-355 trial enrolled 847 patients with locally recurrent inoperable or metastatic TNBC that has not been previously treated with chemotherapy in the advanced setting. Tumor samples were graded for PD-L1 expression based on the combined positive score (CPS), which measures PD-L1 expression on tumor cells, lymphocytes, and macrophages.

Patients were randomly assigned 2:1 to receive pembrolizumab every 3 weeks (*n* = 566) or placebo (*n* = 281) in addition to *nab*-paclitaxel, paclitaxel, or gemcitabine/carboplatin. The co-primary endpoints were overall survival (OS) and progression-free survival (PFS) in 3 patient cohorts defined by PD-L1 expression: CPS ≥10 (*n* = 323), CPS ≥1 (*n* = 636), and the intent-to-treat (ITT) study population (*N* = 847).

Pembrolizumab was associated with a statistically significant 27% improvement in OS in the subgroup of patients with high PD-L1 expression, defined as CPS ≥10 (HR, 0.73; *p* =.0093) (Table 2). Although pembrolizumab was associated with a trend toward improved survival in other subgroups, the benefit did not reach the prespecified threshold for statistical significance.

**Table 2.** Overall survival in patients with locally advanced or metastatic TNBC

Median Overall Survival	Pembrolizumab plus chemotherapy (*n* = 566)	Placebo plus chemotherapy (*n* = 281)	HR (95% CI)	*p* value
PD-L1 CPS ≥10	23.0 months	16.1 months	0.73(0.55-0.95)	.0093
PD-L1 CPS ≥1	17.6 months	16.0 months	0.86(0.72-1.04)	.0563∗
All patients	17.2 months	15.5 months	0.89(0.76-1.04)	NR

∗Prespecified *p*-value boundary of.0171 for statistical significance not met.

Abbreviations: CI, confidence interval; CPS, combined positive score; HR, hazard ratio; NR, not reported; PD-L1, programmed death-ligand 1; TNBC, triple-negative breast cancer.

Pembrolizumab showed a trend toward improved PFS in all groups (Table 3), although the benefit reached statistical significance only in the subgroup of patients with PD-L1 CPS ≥10. In this group, pembrolizumab improved PFS by 34% (HR, 0.66; 95% CI, 0.50-0.88).

**Table 3.** Progression-free survival in locally advanced or metastatic TNBC

Median PFS	Pembrolizumab plus chemotherapy (*n* = 566)	Placebo plus chemotherapy (*n* = 281)	HR (95% CI)
PD-L1 CPS ≥10	9.7 months	5.6 months	0.66 (0.50-0.88)
PD-L1 CPS ≥1	7.6 months	5.6 months	0.75 (0.62-0.91)
All patients	7.5 months	5.6 months	0.82 (0.70-0.98)

Abbreviations: CI, confidence interval; CPS, combined positive score; HR, hazard ratio; PD-L1, programmed death-ligand 1; PFS, progression-free survival; TNBC, triple-negative breast cancer.

Based on these findings, the study authors concluded that CPS ≥10 is a reasonable threshold of tumor PD-L1 expression to define the population of patients with metastatic TNBC who are most likely to benefit from treatment with pembrolizumab in the advanced/metastatic setting.

## Notes

Please note that this content was created for the purpose of summarizing the content of the San Antonio Breast Cancer Symposium 2021 and has not been peer reviewed under the standard policies of *The Oncologist*.
